# Editorial: Industrialization and commercialization in tissue engineering and regenerative medicine: 2022/2023

**DOI:** 10.3389/fbioe.2024.1371633

**Published:** 2024-01-23

**Authors:** Kim S. Siow, Wen Shi, Behnam Akhavan

**Affiliations:** ^1^ Institute of Microengineering and Nanoelectronics, Universiti Kebangsaan Malaysia, Bangi, Selangor, Malaysia; ^2^ Division of Cardiology, Department of Internal Medicine, University of Nebraska Medical Center, Omaha, NE, United States; ^3^ School of Engineering, College of Engineering, Science and Environment, University of Newcastle, Callaghan, NSW, Australia

**Keywords:** skin regeneration, nanofiber, antibaceterial, tissue engineering, bioprinting

Tissue Engineering and Regenerative Medicine (TERM) combines engineering and biological sciences to bring about a paradigm shift in healthcare. The aim of this multidisciplinary discipline is the development of biomimetic scaffolds, cellular treatments, and bioactive compounds to facilitate tissue regeneration. The development of functioning tissues and organs in the laboratory has been driven by progress in materials science, stem cell biology, and biotechnology. This Research Topic contains four articles covering Research Topic from developing new skin regeneration techniques in the pre-commercialization phase to the recognition of tissue engineering commercialization and its impact to the local community in Canada.

In brief, the first article entitled “Weakly acidic microenvironment of the wound bed boosting the efficacy of acidic fibroblast growth factor to promote skin regeneration.” explores the effects of acidic fibroblast growth factor (aFGF) in a weakly acidic microenvironment for skin regeneration. It investigates the enhanced efficacy of aFGF under these conditions, which could have implications for improving skin regeneration techniques.

The second article is titled “Antibacterial, injectable, and adhesive hydrogel promotes skin healing.” It details the development of a novel hydrogel based on carboxymethyl chitosan (CMCS) and polyethylenimine (PEI), focusing on its physical properties, antibacterial abilities, and potential for wound healing applications. The study delves into the preparation, characterization, and *in vitro*/*in vivo* testing of the hydrogel, demonstrating its efficacy in promoting skin regeneration and its possible future use in medical applications.

The third article, “Immune evaluation of granulocyte-macrophage colony stimulating factor loaded hierarchically 3D nanofiber scaffolds in a humanized mice model,” focuses on the immune responses to biomaterials used in tissue regeneration. It examines the effectiveness of GM-CSF loaded 3D radially aligned nanofiber scaffolds in humanized mice models, revealing their potential in tissue regeneration by promoting cell migration and enhancing immune responses.

The final article, titled “Market of tissue engineering in Canada from 2011 to 2020,” presents a comprehensive market analysis of tissue engineering in Canada. It details the growth, trends, and potential of the tissue engineering industry, focusing on various segments like bioprinting, biomaterials, cells and biomaterials, and stem cells. The study emphasizes the significant growth of the industry in Canada, underscoring the contributions of various companies and their impact on healthcare, particularly in addressing infectious diseases and life-threatening disorders.

The papers in this Research Topic highlight key successes and learning points in TERM. For instance, the first paper demonstrates the critical role of pH in enhancing growth factors for skin regeneration, while the second features the creation of an innovative hydrogel system for wound healing. On the other hand, challenges faced in these studies, such as the complexities of immune responses to biomaterials discussed in the third paper, and the marketing barriers and segments outlined in the fourth, provide valuable lessons.

These insights emphasize the importance of overcoming technical and commercialization challenges in TERM to achieve clinical and market success. The articles in this Research Topic collectively underscore the rapid advancements in TERM. They emphasize the necessity of a multidisciplinary approach, blending scientific discovery with engineering innovation for effective clinical translation. This synergy is crucial in developing practical and impactful healthcare solutions. It underlines TERM’s expansive potential, from laboratory research to commercialization, reinforcing its significance in modern medical science and industry.

